# Potential use of saline resources for biofuel production using halophytes and marine algae: prospects and pitfalls

**DOI:** 10.3389/fpls.2023.1026063

**Published:** 2023-06-02

**Authors:** Zainul Abideen, Raziuddin Ansari, Maria Hasnain, Timothy J. Flowers, Hans-Werner Koyro, Ali El-Keblawy, Mohamed Abouleish, Muhammed Ajmal Khan

**Affiliations:** ^1^ Dr. Muhammad Ajmal Khan Institute of Sustainable Halophyte Utilization, University of Karachi, Karachi, Pakistan; ^2^ Department of Biotechnology, Lahore College for Women University, Lahore, Pakistan; ^3^ Department of Evolution Behaviour and Environment, School of Life Sciences, University of Sussex, Brighton, United Kingdom; ^4^ Institute of Plant Ecology, Research Centre for Bio Systems, Land Use, and Nutrition (IFZ), Justus-Liebig-University Giessen, Giessen, Germany; ^5^ Department of Applied Biology, College of Sciences, University of Sharjah, Sharjah, United Arab Emirates; ^6^ Department of Biology, Chemistry and Environmental Sciences, College of Arts and Sciences, American University of Sharjah, Sharjah, United Arab Emirates

**Keywords:** conservation, halophytes, microalgae, non-food biomass, salinity, sustainability

## Abstract

There exists a global challenge of feeding the growing human population of the world and supplying its energy needs without exhausting global resources. This challenge includes the competition for biomass between food and fuel production. The aim of this paper is to review to what extent the biomass of plants growing under hostile conditions and on marginal lands could ease that competition. Biomass from salt-tolerant algae and halophytes has shown potential for bioenergy production on salt-affected soils. Halophytes and algae could provide a bio-based source for lignoceelusic biomass and fatty acids or an alternative for edible biomass currently produced using fresh water and agricultural lands. The present paper provides an overview of the opportunities and challenges in the development of alternative fuels from halophytes and algae. Halophytes grown on marginal and degraded lands using saline water offer an additional material for commercial-scale biofuel production, especially bioethanol. At the same time, suitable strains of microalgae cultured under saline conditions can be a particularly good source of biodiesel, although the efficiency of their mass-scale biomass production is still a concern in relation to environmental protection. This review summaries the pitfalls and precautions for producing biomass in a way that limits environmental hazards and harms for coastal ecosystems. Some new algal and halophytic species with great potential as sources of bioenergy are highlighted.

## 1 Introduction

**Figure 1:**
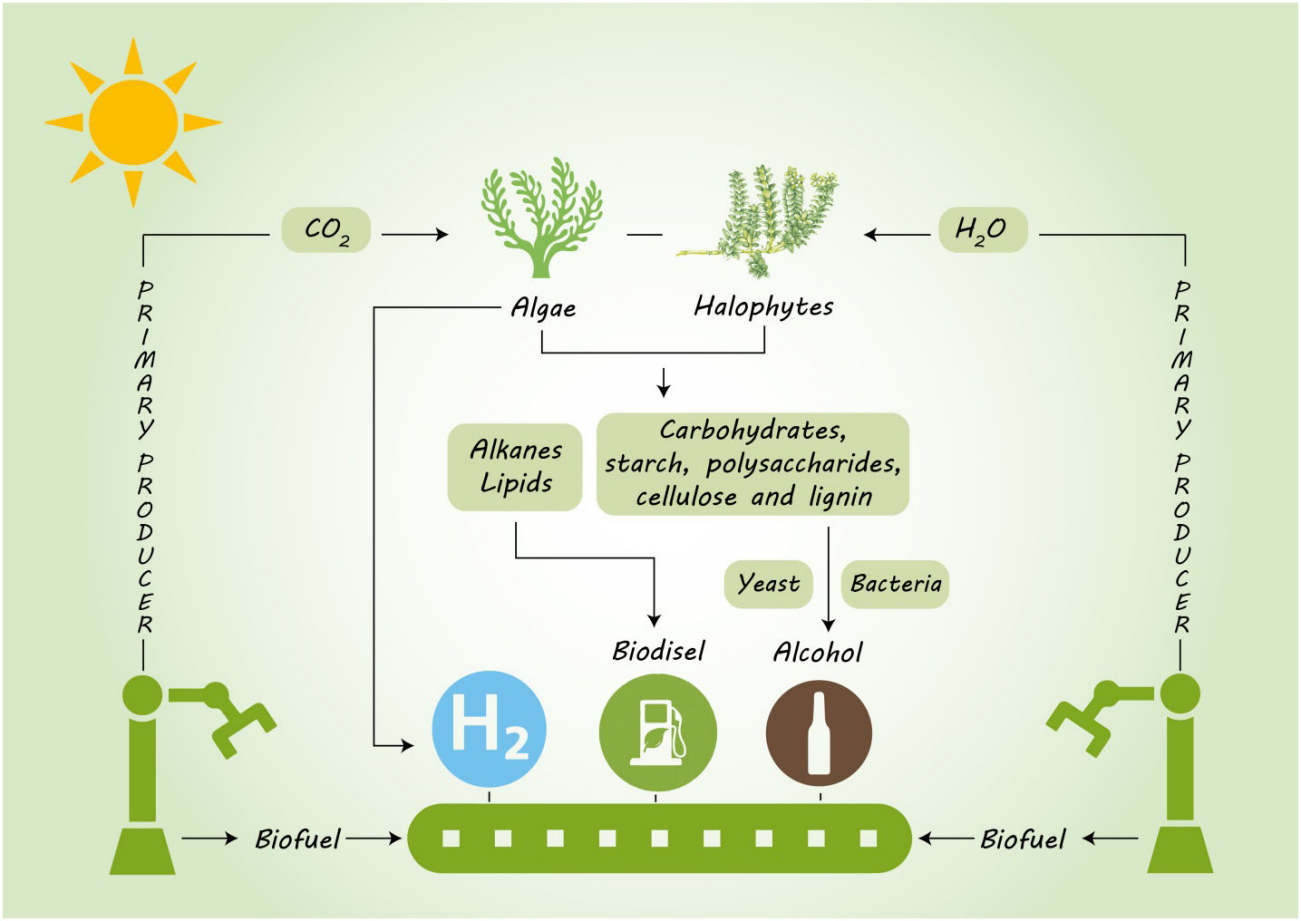
Potential of algae and halophytes as a sustainable biofuel feedstock by using saline land and brackish water.

There is a growing demand to feed the expanding human population and to supply its energy needs without exhausting the biological and physical resources of the planet. Besides food security, clean and renewable energy is also central to achieving at least 20% of the world’s total energy use with renewable resources by 2020, and 32% by 2030. Field crops like sugarcane, corn, soybean and some cereals have so far been the major source of biofuel production, but their use is in direct conflict with their use as food crops (Wang et al., 2020b). Bioenergy production from unconventional non-edible resources offers significant potential as an alternative, but is not without consequences that need careful consideration from a range of environmental, social and economic perspectives (Hasnain et al., 2021). The consequences of bioenergy production depend on biomass conversion technology, types of lands used for annual crops, forest, grassland, or marginal land, the location and level of production, and how these factors integrate with or displace existing land use (Abideen et al., 2015b). This review is based mostly, but not exclusively, on a global scenario of climatic change, and the advantages and pitfalls of using algae and halophytes as non-food bioenergy crops with potential as sustainable bioenergy resources (**Figure 1**)

### 1.1 Sustainable alternative bioenergy technologies: advantages and disadvantages

The global population is expected to exceed nine billion by 2050, with the current demographic trends requiring an average annual increase of 44 million tons of food production (Bologna and Aquino, 2020). Achieving this goal is challenging because of the increasing threat of soil salinization and desertification, which are reducing the amount of arable land and crop yields. It is worth mentioning that nearly 98% of earth’s water is saline (Li et al., 2020b), ~7-10% of the land surface is estimated as salt-affected (Zhang et al., 2020), and although the scourge of soil salinization is spreading worldwide, the situation is worse in arid and semiarid regions (Edrisi et al., 2020). For instance, India has 7 million ha (Mha) of saline lands, Bangladesh 1 Mha, Pakistan 3-6 Mha, and Australia 2 Mha (Rogers et al., 2020). The current estimate of 3 ha of arable land becoming infertile due to secondary salinization every minute indicates the gravity of the situation and a cause of concern requiring remedial steps (Farooqi et al., 2021). The acuteness of this problem is compounded by changes to the global climate that are expected to increase the frequency and severity of temperature extremes, drought in some places, and floods in other regions (Sharif et al., 2021), with adverse effects on crop production.

Energy from fossil fuels is important in sustaining human life on earth, but the long-term availability of coal, oil, and natural gas is uncertain and projected to last only until the middle of the next century (Meyer et al., 2017). In order to search for alternative fuels, numerous feedstocks have been identified. Plant-biomass-based feed stocks are consequently gaining momentum with different potential candidates; e.g. edible (first generation), non-edible (second generation) and various saline-irrigated (third generation) feedstocks (Abideen et al., 2014a). Plant-based first-generation biofuel feedstocks such as sugarcane, wheat, corn, rapeseed and soybean have been used extensively for this purpose, but their use faces opposition due to impacts on the human food chain, while second generation crops such as *Miscanthus* and *Jatropha* require land and water that are needed for food crops (Munir et al., 2021). Production of biofuels from biomass of plants that can grow in saline habitats has consequently emerged as an environmentally-friendly, practical and economical alternative (Abideen et al., 2012). Growth rates of halophytes produce similar yield with saline irrigation compared to conventional crops. For instance, using seawater irrigation of plants of *Salicornia bigelovii* yielded 10 to 20 ton/ha of biomass, equivalent to the yield from conventional crops (Christiansen et al., 2021).

Using salt-tolerant species offers an economic opportunity, as well as a possible mechanism to reduce greenhouse gas (GHG) emissions and enhance energy security without encroaching upon resources (arable land, fresh water) needed for crops for human consumption (Abideen et al., 2015b). Increasing the list of environmentally sustainable sources of biofuel by using feedstocks is a proactive soil-security concept necessary for offsetting land degradation and desertification. Similarly, use of algal biomass can enhance the biological and physical resources of the planet, increase the supply of non-crop biomass to produce clean and renewable energy (such as a feedstock for biodiesel) and help to secure food security by reducing the competition on field grown biomass. Algae are largely non-food resources which do not necessarily need arable land and good quality water as many algal strains grow in seawater (Abideen et al., 2020). In addition, algal production offers remarkably high growth rates leading, for example, to a generation of up to 15 times more oil production per ha than palm, rapeseed or *Jatropha*.

Halophytes and algae are alternative resources for biomass and could be used to reduce the food *via* fuel dilemma. While the practicalities of large-scale production are still questionable, solutions may even open the way for a win-win situation by reducing the problems of insufficient bioenergy supply, excessive greenhouse gas (GHG) emission and uncontrolled desertification. The question remains whether biofuel production fed by halophytes and algae can be maintained with long-term stability. This review focuses on the advantages and disadvantages of biofuel production from algae and halophytes. The review highlights the major advancements achieved through biofuel processing technology to enhance bioenergy production. Technical insights that help to maintain optimal operating parameters for successful operation of biofuel processing from algae and halophytes are summarized. The technology must overcome a number of hurdles before it can compete in the fuel market and be broadly deployed. These challenges include strain identification and improvement, both in terms of oil productivity and crop protection, nutrient and resource allocation and use and production of co-products to improve the economics of the entire system. As far as we are aware, there are no studies on a comparison of algae and halophytes as sustainable sources of energy. The mass-scale cultivation of these potential resources for bioenergy production can help to revolutionized world energy production while avoiding competition in land use for food production. In view of the current situation with the energy-water-food nexus and use of plants for bioenergy, the use of new non-food resources, such as halophytes and algae, requires evaluation. Halophytes and algal species are salt-tolerant organism that can prosper in sea or brackish waters and are feedstocks for fuel and food (fuel-food feedstocks) in developing countries. The suitability of these feedstocks is reviewed and recommendations and solution for their cultivation in saline agriculture highlighted. The aim of this article is to incentivize efficient saline agricultural biomass production. We elucidate the major challenges to the economic production of algal and halophytic biofuels at scale, and provide a focus for the scientific community to address the challenges of moving these feedstocks from promise to reality.

## 2 Algal biomass as biofuel feedstock

Algae, ranging from small, single-celled microalgae to multi-cellular macroalgae, occupy a variety of habitats from damp places to bodies of fresh or sea water [36-38] and present a wide range of species that could be cultivated. High oil content; in some cases almost 80% of cell weight, together with the high growth rates with biomass doubling in periods as short as 3.5 h have generated interest in using algae as biofuel feedstock (Jazie et al., 2020). Oil production from algae can exceed that from the oilseed crops such as rapeseed, canola (Petrie et al., 2020), *Jatropha* (Kumar et al., 2017) and karanja (*Pongamia pinnata*) (Tıpırdamaz et al., 2020). Growing algae on a large scale has the benefit of removing greenhouse gases by consuming CO_2_ for photosynthesis during growth (Gharbia et al., 2019). By utilizing their high photosynthetic ability, algae appear an attractive energy feedstock among renewable resources, for rapid generation of carbohydrates and lipids. There are, in addition, other possible benefits such as use for human food (albeit on a limited scale) or as a source of byproducts of commercial interest (Roostaei et al., 2018). Like other plants, growing algae requires optimizing water, nutrients, light, temperature and pH (Hasnain et al., 2023). The following issues require consideration and further research.

### 2.1 Culturing space

Availability of suitable land is the primary limiting factor for bioenergy development from algal feedstock The largest micro-algal commercial production unit covers 750 ha using an open pond system, but even this facility is insufficient to meet local biofuel demands (Randhir et al., 2020). Although, open ponds are easier to build and operate than closed systems, a large quantity of water is evaporated. Other constraints that limit algal production are: low light penetration, poor carbon dioxide diffusion from the atmosphere, inefficient stirring causing poor mass transfer, and contamination by microbes and other algal species (Talaei et al., 2020). Proper mixing systems are required if sedimentation is to be avoided and light utilization maximized (Fong-Lee et al., 2020). Closed systems such as photo bioreactors are used to overcome problems of open pond cultivation systems. A photo bioreactor (PBR) is a closed vessel and energy is supplied through electric lights (Zhao et al., 2021). PBRs are classified as flat plate, tubular or columnar on the basis of their illuminated surface and stirred, bubble-column or airlift depending on how mixing is achieved (Sero et al., 2020). So, a PBR should have a highly transparent surface, good uniform illumination, low mutual shading, quick mass transfer of carbon dioxide and oxygen and should attain high growth (Amaral et al., 2020).

### 2.2 Photobioreactor (PBR) design improvements (Data collection and modeling)

Experimental evaluation and consecutive model development are used to predict the behavior of actual and expected algal growth at different culturing conditions, which can be optimized *in situ* (Sheehan et al., 2020).

### 2.3 Light utilization and mixing of algal culture

As light is the source of energy, it needs optimization for biomass productivity (Munir et al., 2022). High productivity of algal culture requires annual average sunshine of 2,500-5,000 lux (its intensity, spectral quality and photoperiod), and temperatures in the range of 18-24°C. Flat plate PBRs are more efficient in utilizing sunlight than other PBRs because of their flat surface area. Light utilization can be optimized using panels of tubes and supplementary light from light emitting diodes (LEDs) using fiber optics (Alzahrani et al., 2020). About 1.5% photosynthetic efficiency has been achieved using the open pond method with 21 ton/ha algal productivity, while with tubular PBRs photosynthetic efficiency was 3% yielding 41 ton/ha algal biomass (Clements et al., 2020). The highest photosynthetic efficiency achieved with flat panel PBRs has been 5% with 64 ton/ha algal productivity (Negi et al., 2020). For naturally illuminated PBRs, the orientation with respect to the sun is highly critical if photosynthetic efficiency is to be maximized. To gain maximum light; at latitudes, above 35° N, an orientation of east/west is preferable over north/south for optimal algal productivity (Sivakaminathan et al., 2020). The effects of self-shading in PBRs are reduced by using thinner algal cultures. Circulation is an important step to maintain proper mixing of algal cells in suspension, remove thermal stratification, optimize the distribution of nutrients, boost gas-liquid mass transfer, and stop oxygen accumulation (El Shenawy et al., 2020). Moving algal cells between the illuminated surfaces and dark regions to induce periodic light/dark cycles, is also very important for maximum growth (Alami et al., 2021). By using circulatory apparatus, with cultures of *Spirulina platensis*, 0.5 g/L/day algal cell productivity was obtained, which was assumed to be a high value by the researchers (Serrà et al., 2020).

Algae are an attractive energy source but important questions still exist about the sustainability of this technology on a large scale. Two particularly important questions concern the method of cultivation and the type of alga to be used. Resurreccion et al. (2012) combined elements of life cycle analysis (LCA) and life cycle costing (LCC) to evaluate open pond systems (OPs) and horizontal tubular photobioreactors for the cultivation of freshwater or brackish-to-saline water algae. According to the LCC, all four systems are currently financially unattractive investments, though OPs are less so than PBRs (Sun et al., 2019). Salt-tolerant species deliver better energy and GHG performance and higher profitability than fresh water species in both OPs and PBRs. Sensitivity analyses suggest that improvements in critical cultivation parameters (CO_2_ utilization efficiency or algae lipid content), conversion parameters (anaerobic digestion efficiency) and market factors (costs of CO2, electricity, and sale prices for algae biodiesel) could alter these results (Muhammad et al., 2021).

### 2.4 Algal production cost

The overall production cost of algae depends on the selection of algal strain, type of PBR and of biomass production technology; a major cost is that of construction if the installation is large (Ma et al., 2017). The cost of production can be reduced by using flue gases instead of CO_2,_ which is very expensive, and using wastewater instead of adding minerals or nutrients to growth media (Roostaei et al., 2018). More than 150 ton/ha/year algal biomass can be produced with low labor costs, using flue gases as carbon source and wastewater as growth medium (Norsker et al., 2011). By using flat panel tubular PBRs, the production cost can be reduced to €0.70/kg or €0.68/kg as compared to open pounds where the cost cannot be reduced below €1.28/kg (Negi et al., 2020; Oostlander et al., 2020). Using arable land and good quality irrigation water is not advisable, but salt-affected/marginal land, including deserts, together with saline/waste water may be used subject to the availability of suitable strains of algae (Amiri and Ahmadi, 2020).

### 2.5 Growth conditions

Biofuel production depends upon optimizing growth conditions for any species. Good growth in open ponds can be achieved but, generally, not over a whole year, because of short days, low light intensity and temperature during winter, the more so in cold regions (Jacob et al., 2020). However, higher oil production under stressed than optimal conditions has been reported by some researchers (Juneja and Murthy, 2017; Bélanger-Lépine et al., 2018; Tan et al., 2020), but needs assessment. With photo-bioreactors, it is important that mixing is optimized so that algal cells are transferred between light and dark phases; this has allowed the successful use of very high light intensities (Severes et al., 2017). During photosynthesis, photons are used to synthesize biochemical components in the algae and in different light conditions, a change in photon flux can bring about a noteworthy change in the biochemical compositions of algal cells (Naira et al., 2020). A higher intensity or length of light energy triggers the accumulation of storage lipids (MUFAs) used in biodiesel production, while a lower intensity stimulates the accumulation of structural lipids (PUFA) (Brindhadevi et al., 2021). Carbohydrate content was enhanced from 16.3 to 22.4% in *Scenedesmus obliquus* by the exposure to high light intensity (Ho et al., 2017). Physicochemical properties of algae also change with increasing or decreasing temperature. **Table 1** shows the effect of different light intensities and temperature on lipid content and fatty acid profile.

**Table 1:** The impact of variable light intensities, change of temperature and pH on lipid accumulation and fatty acid profiles on the basis of % dry weight.

Algae	Light intensities	Algae response	References
*Chlorella vulgaris*	2700 lx	lipid 19%	(Azizi et al., 2020)
	3300 lx	lipid 13%	
*Chlorella vulgaris*	24 h photoperiod	Lipid production increased	
*Pavlova lutheri*	High light intensities	Lipid production increased	
*Isochrysis galbana, Nannochloropsis oculata*	High light intensities	Lipid production increased	
*Scenedesmus* sp.	250–400 µmolm^2^ s −1	Lipid production increased	
*Isochrysis galbana*	Shorter light/dark regime	PUFA’s increased	
*Selenastrum capricornutum*	Dark treatment	Increase in 18:3 and decrease in 18:1	
Algae	Temperature (°C)	Response	References
*Nannochloropsis oculata*	Shift – 20 to 25	15% increase in total lipid	(Wei et al., 2015)
*Chaetoceros* sp.	25	16.8% increase in total lipid	
*Ochromonas danica*	15 to 30	Increase in total lipids	
*Nannochloropsis oculata*	> 30	Polar lipid increased	
*Nitzschia laevis*	15 to 23	TAG increased	(Touliabah et al., 2020)
*Dunaliella salina*	30 to 12	UFA’s increased	
*Synechococcus Lividus*	55 to 38	16:1 and 18:1 increased	
*Selenastrum capricornutum*	25 to 10	C 18:1 increased	
Algae	Optimal pH	Response	References
*Chlorella* sp.	8	Lipid content 23%	(Qiu et al., 2017)
*C. vulgaris*	7.5	53.43	
*Chlorella protothecoides*	6.5	3.75 g/l lipid yield	
*Pavlova lutheri*	8.0	35% lipid content	
*Nannochloropsis salina*	8.0–9.0	21.8	
*T. suecica*	9.0	lipid content increased	
*Chlorella vulgaris*	7.0, 8.0, 9.0, and 10.0.	No change	(Nouri et al., 2021)
	6.0, 7.0, 9.0	No change	
*Chroococcus minor*	9.0	22	
*B. braunii*	6.5	2.2 gm^−2^/d lipid productivity	

### 2.6 pH effects

Physiological and biochemical functions for optimal growth of most algal species require maintenance of neutral pH in the growth medium (Correa et al., 2020). The relationship between CO_2_ concentration and pH in algal growing system is complex (Alzahrani et al., 2020). Increasing internal CO_2_ concentration can lead to higher photosynthetic efficiency but it can also decrease pH; these two factors and their antagonism can alter algal physiology and biomass production. An increase in pH of growing media can be favorable for inactivation of harmful pathogens in open-type growth chambers, but can also inhibit growth (Almutairi and Toulibah, 2017). Growth medium pH above 9 generally creates difficulty for the utilization of $HCO_{3}^{-}$ and $CO_{3}^{2}$ for maintaining internal CO_2_ although there are some resistant species that can survive at pH above 9, but at the cost of metabolic disturbances and compromises in productivity (Alami et al., 2021). In addition to low capacity to absorb internal CO_2_, elevated pH interferes with the cell’s ability to maintain activity of carboxylation *via* Rubisco, which reduces photosynthetic rate (Li et al., 2020a).

High pH may convert ammonium ions to free ammonia which in quantities such as 34 and 51 gm^3^ (at pH 9.5 and 20-25 C) inhibited the rate of photosynthesis in three micro-algal cultures by 50 and 90%, respectively (Galès et al., 2020). The presence of ammonia also creates problems in sunshine, especially during summer when light intensities may raise pH of the growing medium and ultimately inhibit photosynthesis. Elevated pH can also alter membrane transport processes, metabolic function and uptake of trace metals consequently affecting photosynthesis and growth of algae (Miyauchi et al., 2020). Under elevated pH, flocculation of some microalgal cultures can occur, negatively impacting light absorption, photosynthesis and nutrient uptake. If wastewater is being used for algal culture, pH above 8.3 has been reported to inhibit growth of aerobic bacteria: high pH tolerating algal strains can be used in high pH waste water for cultivation (Vadlamani et al., 2017).

#### 2.6.1 Water availability

The production of algal biomass, either in closed photo-bioreactors or open ponds, and its conversion to biofuels, consumes a considerable amount of water (Vu et al., 2020). The availability of fresh water is limited especially in areas where productivity of algae is potentially high - i.e. regions with high year-round solar radiation (Mayer et al., 2020). Algal growth and subsequent processing likely cause substantial water pollution (Rahman et al., 2020). High-value food crops have preference for cultivation with fresh water in arable lands, necessitating exploring the potential of biofuel feedstock from those species that can grow optimally and complete their life cycle in marginal/saline lands irrigated with low quality/brackish water (Fork et al., 2020). **Table 2** shows the salt tolerance of algal strains.

**Table 2:** Salinity and heat resistance and their interactive effect on growth responses of algae strains.

Algae species	Salinity levels	Temperature °C	Growth rates	References
*Dunaliella tertiolecta*	33 to 59 g/L	23	1.9696 g/L	(Morales-Sánchez et al., 2020)
*Chaetoceros calcitrans*	3%	30	0.28 µl/d	
*Chlorella* sp.	2.5%	25	0.37 µl/d	(Gour et al., 2020)
*Fucus vesiculosus.*	5 psu	4-10	0.007 g/g.d	(Zamani-Ahmadmahmoodi et al., 2020)
	35 psu	15–20	0.024 g/g.d	
*Nannochloropsis oculata*	15–55 g/L	26	0.078-0.282/d	
*Desmodesmus* sp.	15 g/L	25	5.35 g/L	(Chen et al., 2020)
*Ulva prolifera*	14–32	5–32	10.6–16.7%/d	(Bews et al., 2021)
*Hypnea cervicornis*	25	20–25	5.37%/d	(Vo et al., 2020)
*Shewanella* sp.	0–7%	30	0.04 to 0.36 g/L	
*Chaetomorpha* sp.	3.4–90.0	20.1–40.9	60%/d	

Biological desalination is an innovative technology in which salts are absorbed by salt-tolerant organisms. For example, *Scenedesmus obliquus* is a fresh-water alga with a high tolerance of salts and the capacity to remove NaCl from (0.18- 1.4 g/L) when salinity increased from 2.8- 8.8 g/L (El-Katony and El-Adl, 2020). Salinity also enhances hydrocarbon content in algae: *S. obliquus* treated with 8.8 g/L NaCl increased its lipid content (21%) while removing 2.5 g/L NaCl and achieving the highest desalination rate (30%) within 30 min contact time (Wei et al., 2020). The rate of evaporation is another limiting factor in any open pond system and one that will increase when days are long and intensity of light is high in hot regions. The lost volume of water should be made up with fresh water but this might burden supply of this commodity if scarce (Prudkin-Silva et al., 2021). If fresh water is not added, the salinity of the medium may consequently rise to a level too high for growth of even highly salt-resistant strains. A solution may be found in frequent renewal of evaporated water which could, however, pose other problems, as any discharged saline water may still have unused nutrients (Khuram et al., 2019) such as phosphorus and nitrogen creating a disposal challenge downstream, a potential additional cost and raising questions about environmental sustainability. Use of wastewater as a source of water has been advocated to attain a double benefit of biofuel production as well wastewater treatment (Juneja and Murthy, 2017).

### 2.7 Nutrient requirements

The elemental composition of algae with empirical formula CH_1.7_O_0.4_N_0.15_P_0.0094_ will generally fluctuate with environmental conditions and nutrient status of the strains and growth medium. The essential nutrients (N, P, and in some cases Si) should be provided in adequate amounts for optimal growth (Tan et al., 2020). Approximately 50-80 kg of nitrogen and 5 kg of phosphorus are required to produce one ton of algal biomass. Commercial production of algal biofuels would hence need large quantities of these elements and maybe of other nutrients. For instance, if saline ground water is used, the medium may also require potassium. Algal biomass production under the circumstances can hence be competing with the edible plants for nutrient requirements (El-Katony and El-Adl, 2020). Moreover, production of fertilizers needed for algal growth is at an environmental cost due to use of energy and emission of considerable amounts of the greenhouse gases carbon dioxide, nitrous oxide and methane (Yuasa et al., 2020). It has been reported that ~45% energy input in algal culture is in the form of nitrogenous fertilizer; each kg of this fertilizer produced from natural gas, which is also depleting, generates about 2 kg of CO_2_.

Phosphorus is not a renewable resource and, at current rates of mining, global phosphate rock reserves are likely to be finished in 50-100 years (Dubey and Dutta, 2020). Taking into consideration the nutrient requirements, which may vary with species, a study found that meeting the demand of algal bioenergy to substitute 5% of the fuel used annually for transportation in the United States would require 44-107% of the total nitrogen and 20-51% of the total phosphorus requirement of the country. In the natural ecosystem, the phosphorus and nitrogen requirements are met to a great extent by dead bodies of animals but in artificial systems these have to be supplemented at a cost to the purchaser. Wastewater can be used as a source of nutrients to attain a double benefit of biofuel production as well as wastewater treatment and pollutant removal (Juneja and Murthy, 2017). *Acutodesmus obliquus* culture consumed 175 mg/g/day nitrogen and 1.5 mg/g/d phosphorus from swine wastewater having 5.2% salinity with 1923 mg/L/d chemical oxygen demand (El-Katony and El-Adl, 2020). At 11 ppt of salinity *Picochlorum atomus* nutrient uptake was four times higher than controls, 34 mg/L/d uptake of nitrogen and 1.3–2.4 mg/L/day uptake of phosphate (Zhang et al., 2018). At 3.2% salinity, *Spirulina platensis* removed 80% nitrogen, 93% phosphate and 90% COD with 15.69, 1.03, and 90.24 mg/L effluent concentration respectively (Koech et al., 2020).

#### 2.7.1 Improved growth capacity through increased photosynthetic efficiency

The production of any biofuel is dependent on the efficiency of the metabolic pathways that lead to accumulation of storage compounds, such as lipids and starch, as well as on the ability to produce large amounts of biomass rapidly. Experiments with small- and large-scale microalgal photobioreactors and molecular research in photosynthetic efficiency have revealed several factors that can limit biomass accumulation. One important consideration is the intensity of light at which a given strain of microalga reaches its maximum growth rate, which corresponds to the maximum photosynthetic efficiency and is usually around 200 to 400 µmol photons m^-2^ s^-1^ for most species (Lee et al., 2002). Light intensities above the maximum photosynthetic efficiency actually reduce the growth rate, a phenomenon known as photoinhibition. Photosynthetically active radiation intensities from sunlight can exceed 2,000 µmol photons m^-2^ s^-1^ during midday. Consequently, most microalgae will not grow at maximum efficiency during most of the day.

Microalgae are considered great model organisms to study photosynthetic efficiency, and several attempts have been made to improve the photosynthetic efficiency and/or reduce the effects of photoinhibition on microalgal growth (Melis, 2009). Much of this work has been focused on reducing the size of the chlorophyll antenna or lowering the number of light-harvesting complexes to minimize the absorption of sunlight by individual chloroplasts. This approach may seem counterintuitive, but this strategy may have two positive effects; first, it permits higher light penetration in high-density cultures and second, it can allow a higher maximum rate of photosynthesis due to the fact that the cells are less likely to be subjected to photoinhibition since their light-harvesting complexes absorb less light (Priyadharsini et al., 2022). Earlier, research relied on random mutagenesis strategies to generate mutants with fewer or smaller chlorophyll antennae, but a recent publication used an RNAi-based strategy to knock down efficiently both LHCI and LHCII in *C. reinhardtii* (Mussgnug et al., 2007). This strategy can most likely be applied to many different microalgae more easily than a random mutagenesis approach. It seems clear that manipulation of light-harvesting complexes can lead to increased biomass productivity under high light in controlled laboratory conditions. However, it remains to be seen how well these mutants will perform in larger-scale cultures with more varied conditions and perhaps with competition from wild invasive microalgal species. In one study of algal antenna mutants, no improvement in productivity was observed with outdoor ponds (Mussgnug et al., 2007). However, they also did not observe any improved productivity in laboratory cultures. With more research, it should become clear whether the current approach can be successfully applied to increase biomass production.

### 2.8 Pollution of land and aquatic system

Production of wastewater derived from many anthropogenic activities such as industry, agriculture and domestic use is a major environmental issue and a threat to water security. About half of the global waterbodies such as the lakes, rivers and seas have been contaminated by domestic and industrial wastewaters; it is essential to treat and remediate wastewater so that it could be recycled and reused (Aron et al., 2021). Discharge of residual nutrients from algal cultures could have a negative impact on land and aquatic systems and the natural flora of an area. Ecological consequences that can occur includes decrease in biodiversity, changes in species richness and altered fitness of other living organisms. Toxic discharge and accumulation may create problems for plant agriculture and restoration can be a costly long-term process, depending upon the extent of damage due to effluents (Liu et al., 2017). Introducing production of suitable algal strains that would scavenge harmful excess nutrients could, however, help in reversing the damage. For instance, algal turf scrubber (filtering device) can capture 70-100% of phosphorus runoff and 60-90% of nitrogen from manure effluents (Aston et al., 2018). Wastewater from municipalities, agriculture and industry could provide cost-effective and sustainable support for the use of algae for biofuels (Tan et al., 2020) In addition, there is also potential for combining wastewater treatment, such as nutrient removal, with biofuel production. The following are three examples; at 35 g/L NaCl, *Potamocorbula laevis*, *A. nodosum* and *F. vesiculosus* accumulated copper from growing medium (Vo et al., 2020); in saline wastewater (2.6% salinity), by bio-assimilation and adsorption *Chlorella* sp. removed 99% of amoxicillin; and under 171 mM NaCl, *S. obliquus* biodegraded 93.4% of levofloxacin (Leng et al., 2020).

#### 2.8.1 Environmental aspect of microalgae cultivation

Microalgae introduced into new environments also have the potential to become invasive species. There are an estimated 1–10 million algal species on earth, with the majority being microalgae (ElFar et al., 2022). Microalgae that are native or introduced to an area and become invasive are often referred to as harmful algal blooms (HABs). HABs are species of phytoplankton that cause negative effects on human health (through the production of toxins), impact living marine sources (wild and cultivated fish), impact tourism and recreation of coastal waters (through ‘red tides’) and damage marine ecosystems by creating anoxic areas that kill marine life (Anton et al., 2019). There are approximately 80 toxic and 200 noxious microalgal species involved in HABs out of a total of 4000 described marine planktonic microalgae (**Table 3**). Research indicates that the rise in HABs shows the signs of a global epidemic (Zedler and Kercher, 2004). Whether this recognition is the result of an increase of scientific awareness of toxic algal species, utilization of coastal waters for aquaculture, cultural eutrophication of waters, unusual climatalogical conditions or the transport of dinoflagellates by ships ballast water or shellfish stock is unclear. The invasion patterns of microalgae are dependent on human vectors and subsequent adaptation of the algae to their new environment (Gressel et al., 2013). Anthropogenic nutrient enrichment of coastal areas has also been linked to HAB events around the world. Microalgal genera or species proposed for biofuel production that have had HAB incidents include *Amphora*, *Nitzschia*, *Pseudo-nitzschia* and *Prymnesium parvum*. It has been suggested that locating algal biofuel production plants close to seawater will remove the need for fresh water resources and increase their sustainability (Nyström, 2017). However there is little discussion on the ecological impacts resulting from an accidental introduction of a microalgal biofuel species into the surrounding environment. **Table 4** shows the number of chemicals that are potentially toxic for water and human food materials. Documentation and more studies are required to protect wildlife from HABs, the effects of red tides and freshwater cyanobacterial blooms in the future (Müller et al., 2020). Reducing fertilizer use, improving animal waste control, and sewage treatment should also reduce the population of toxic algal blooms. **Tables 5**, **3** listed various methods to control harmful algal blooms from water water and other water resources.

**Table 3:** Organisms used for the removal of harmful algal blooms (HABs) from their populations.

Predatory bacteria	Mode of action	Major host	References
***Bacillus* sp.**	Cell-to-cell contact mechanism	*Aphanizomenon Flosaquae*	(Jeon et al., 2017)
***Bacillus* sp.**	Production of extracellular product	*M. aeruginosa*	
***Bacillus cereus***	Secretion of cyanobacteriolytic	*Microcystis aeruginosa*	
***Bacillus* sp.**	Secretion of algalytic substance	*Phaeocystis globosa*	
***Brachybacterium***	Produce secondary metabolites	*A. catenella*	
***Cytophaga***	Direct contact	*Microcystis aerugenosa*	
***F. flexilisi*, *F. sancti***	Inhibition of glycolate dehydrogenase Electron transport & nitrogenase activity	*Oscillatoria williamsii*	
***Bdellovibrio*-like bacteria**	Varese Penetration	*Microcystis aeruginosa*	
***M. fulvus***	Entrapment	*Phormidium luridum*	
***Pseudomonas fluorescens***	Indirect attack by alga-lytic substances	*Heterosigma akashiwo*	
***Halobacillus* sp.**	Bio-flocculation	*Microcystis aeruginosa*	
Zoonplankton			
***Daphnia ambigua***	Grazing	*Microcystis aeruginosa*	(Richards, 2019)
***Daphnia hyaline***	Grazing	*Chlorella*	
***D. galeata***	Grazing	*Scenedesmus*	
***Cyclops* sp.**			
***Eudiaptomus gracilis***	Grazing	*Chlorella*	
***Eudiaptomus gracilis***	Grazing	*Microcystis aeruginosa*	
***Cyclopoid copepods***	Grazing	*Anabaena, Microcystis* and Planktothrix species	
Algae			
***Ankistrodesmus falcatus***	Bioflocculation	*Chlorella vulgaris*	(Jalgaonwala, 2020)
***Scenedesmus obliquus***	Bioflocculation	*Chlorella vulgaris*	
***Tetraselmis suecica***	Bioflocculation	*Neochloris oleoabundans*	
***Poterioochromonas***	Grazing	*Microcystis aeruginosa*	
Fungi			
***Trichaptum abietinum***	Direct attack	*Microcystis aeruginosa* & *Microcystis flosaquae*	(Sun et al., 2018)
***Lopharia spadicea***	Direct attack	*Microcystis aeruginosa*	
***Irpex lacteus*,** ***Trametes hirsute Trametes versicolor &Bjerkandera adusta***	Direct attack	*Microcystis aeruginosa*	
Cyanophage			
**SM-1**	Species-specific interaction	*M. aeruginosa*	(Xu et al., 2020a)
**SM-2**	Species-specific interaction	*M. aeruginosa*	
**Ma-LBP**	Species-specific interaction	*M. aeruginosa*	
***Cyanostyloviridae***	Species-specific interaction	*Lyngbya majuscule*	
**Ma-LMM01**	Species-specific interaction	*M. aeruginosa*	
**S-PM2**	Species-specific interaction	*Synechococcus*	
**MaMV-DC**	Species-specific interaction	*M. aeruginosa*	
**MaCV-L**	Species-specific interaction	*M. aeruginosa*	
**SAM-1**	Species-specific interaction	*Broader host range*	
***Myoviridae***	Species-specific interaction	*M. aeruginosa*	
***Siphoviridae***	Bursts and virus lytic cycle	*C. raciborskii*	
**Ma-LEP**	Mechanical stiffness	*M. aeruginosa*	
Fish			
***Hypophthalmichthys molitrix***	Grazing	*Microcystis aeruginosa*	(Gu et al., 2021)
***Aristichthys nobilis***	Grazing	*Microcystis aeruginosa*	
***Hyriopsis cumingii***	Ingestion and digestion	*Microcystis aeruginosa*	
***Oreochromisni loticus***	Ingestion and digestion	*Microcystis aeruginosa*	

**Table 4:** Potential biotoxin producers and human health implications of algal biomass.

Algae	Toxins	Human health implication	References
***Alexandrium catenella*, *A. minutum complex*, *A. ostenfeldii*, *A. tamarense***	Saxitoxins, gonyautoxins	PSP	(Pang et al., 2017)
***Chattonella antiqua*, *C. marina***	Breve-like	NSP	
***Coolia monotis***	Uncertain	Uncertain	
***Dinophysis acuta*, *D. acuminata***	Okadaic acid; dinophysis toxin 2 (DTX2); pectenotoxin 2	DSP	
***G. mikimotoi* complex**	Breve-like	NSP, respiratory distress	(Niu et al., 2021)
***Gyrodinium galatheanum***	Breve-like	NSP	
***Heterosigma akashiwo***	Ichthyotoxic	Ass. with peppery taste	
***Ostreopsis siamensis***	Uncertain	Uncertain	
***Prorocentrum lima***	Okadaic acid; DTX1,4; diol esters	DSP	
***Protoceratium reticulatum***	Yessotoxin	Uncertain	(Zingone et al., 2020)
***Pseudo-nitzschia australis*, *P. delicatissima*, *P. fraudulenta*, *P. multiseries*, *P. pseudodelicatissima*, *P. pungens*, *P. turgidula***	Domoic acid	ASP	
***Fibrocapsa japonica***	Ichthyotoxic	None	

**Table 5:** Various methods to control harmful algal blooms from water resources.

Methods	Techniques	Advantages	Limitations	References
**Chemical methods**	Metals (Fe, Cu, Ca & Al)	Low cost and High residence time	Toxicity against non-target species and Accumulation in the environment	(Li et al., 2020a)
	Photosensitizers (hydrogen peroxide, phthalocyanines and titanium dioxide)	Low cost and Degradability	Risky manipulation and Coloration	
	Herbicides (diuron, endothall, atrazine and simazine)	Low cost and High residence time	Release of toxins	
**Physical methods**	Ultrasound techniques	Low impact on ecosystems and Contamination free	To be confirmed at up-scaled levels	(Kong et al., 2019)
	UV irradiation	Eco-friendly and Contamination free	High energy consumption and To be confirmed at up-scaled levels	
	Membrane filtration technology	Well-established technology and High stability	High cost	
	Adsorption	Eco-friendly and Contamination free	Costly and To be confirmed at up-scaled levels	
**Biology methods**	Aquatic plants	Technically simple reactor	Affect biodiversity and Deteriorate eutrophication	(Zerrifi et al., 2018)
	Aquatic animals	User-friendly and Environmentally sound	It will not work in oxygen-poor conditions, Affect biodiversity and Poor efficiency	
**Combined technologies**	Microorganisms	High specificity and High efficiency	High cost and To be confirmed at up-scaled levels	(Park et al., 2019)
	Ultrasonic radiation and jet circulation to flushing	High efficiency	High cost and To be confirmed at up-scaled levels	
	Combination of uniform design with artificial neural network coupling genetic algorithm	High efficiency and Low cost	To be confirmed at up-scaled levels	

### 2.9 Biomass to biofuel conversions

**Figure 2:**
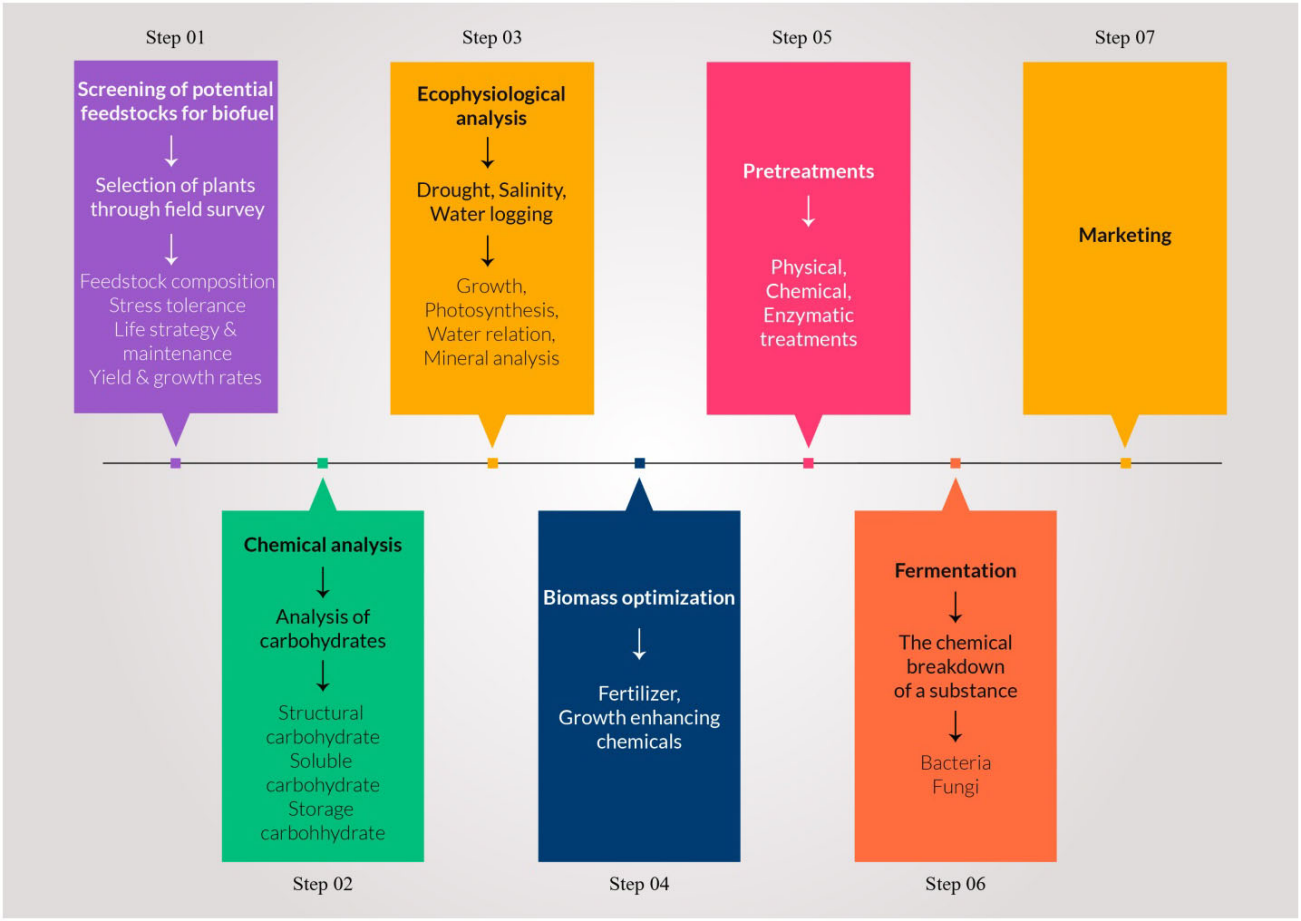
Different key steps of biomass to biofuel conversion form wild halophytic plants from screening to ethanol yield.

Algal cultures are very dilute in nature necessitating dewatering, to produce an algal cake that can be readily handled manually or mechanically for conversion to biofuel (**Tables 6**, **7** and **Figure 2**). However, dewatering is expensive if done by current methods, accounting for approximately 20-40% of the energy required (Tan et al., 2020). Currently, commercial dewatering methodologies include centrifugation, flocculation, coagulation, flotation and sedimentation. Other harvesting techniques such as electrophoresis, electro-flotation, and ultra-sonication are used less frequently but require either prohibitive quantities of energy or harmful chemicals (Almomani, 2020).

**Table 6:** Potential of different biofuels feedstocks on the basis of their oil yield for sustainable energy production.

Species	Oil (%)	References
Soybean	20	(Giuffrè et al., 2020)
Palm oil	30	
Coconut	63	(Suryani et al., 2020)
Rapeseed	38	(Konur, 2021)
Sunflower	25	(Subaşı et al., 2020)
Peanut oil	45	(Dun et al., 2019)
Olive oil	45	(Giuffrè et al., 2020)
Cottonseed	18	(Riaz et al., 2021)
Halophytes		
*Salicornia bigelovii*	30	(Zapata-Sifuentes et al., 2021)
*Cressa cretica*	23	(Afshari and Sayyed-Alangi, 2017)
*Suaeda salsa*	22	(Kefu et al., 2003)
*Haloxylon stocksii*	23	(Abbas et al., 2022)
*Kosteletzkya virginica*	30	(Ruan et al., 2008)
*Atriplex rosea*	13	(Abideen et al., 2015b)
*Ricinus communis*	55	(Salimon et al., 2010)
*Descurainaia sophia*	44	(Mokhtassi-Bidgoli et al., 2022)
*Suaeda torreyana*	25	(Arias-Rico et al., 2020)
*Crithmum maritimum*	40	(D’Agostino et al., 2021)
Algae		
*Botryococcus braunii*	25	(Cheng et al., 2013)
*Chlorella* sp.	28	(Munir et al., 2022)
*Crypthecodinium cohnii*	20	(Li et al., 2015)
*Dunaliella salina*	20	(Yilancioglu et al., 2014)
*Nannochloropsis* sp.	31	(Pal et al., 2011)
*Neochloris oleoabundans*	35	(Tao et al., 2019)
*Nitzschia* sp.	45	(Sahin et al., 2019)
*Schizochytrium* sp.	50	(Ren et al., 2014)

**Table 7:** List of halophytes used for lignocellulosic biomass cellulose, hemicellulose and lignin for bioethanol production.

Species	Cellulose (%)	Hemicellulose (%)	Lignin (%)	References
*Calotropis procera*	12	11	5	(Narayanasamy et al., 2020)
*Suaeda monoica*	10	11	2	(Abideen et al., 2011)
*Panicum virgatum*	45	13	12	
*Suaeda fruticose*	8	21	4	(Abideen et al., 2012)
*Phragmites karka*	26	29	10	
*Arthrocnemum indicum*	11	13	7	(Munir et al., 2020)
*Sporobolus ioclados*	15	30	2	
*Desmostachya bipinnata*	26	24	6	(Abideen et al., 2022)
*Urochondra setulosa*	25	25	6	
*Aeluropus lagopoides*	26	29	7	(Attia-Ismail, 2016)
*Tamarix indica*	12	24	3	
*Cenchrus ciliaris*	22	23	7	(Zheng et al., 2007)
*Eleusine indica*	22	29	7	
*Salsola imbricate*	9	18	2	(Abideen et al., 2011)
*Lasiurus scindicus*	24	29	6	

Filtration using a suction pump with a filter of some sort can be used for dewatering. The advantage over other methods is that algal suspensions of low density can be harvested with very high efficiency. The main problem is clogging of the filter by the algae being harvested (Cha et al., 2020); this has to be tackled by frequent backwashing. Dewatering by centrifugation uses a centrifugal force that is a few orders of magnitude higher than the force of gravity (Hua et al., 2020) but has to be stopped periodically (batch mode) for the solids to be removed (Kong et al., 2020). Efficiency and reliability of centrifugation techniques are high, but so are the operating costs often negating the efficiency of the method. If good quality algae are to be continuously produced, continuous centrifugation (by solid ejecting-type or nozzle-type disc centrifuges) is recommended. These centrifuges, which are suitable for all microalgae, can be cleaned easily and sterilized. However, their cost of operation needs be compared with the value of the end product (He et al., 2020). Harvesting micro-algal biomass by chemical coagulation and flocculation is the most economical of the methods available. The advantage of chemical coagulation/flocculation is that large culture volumes can be collected and the methodology applied to a wide variety of species. Microalgal suspensions can be concentrated 20-100 times by this harvesting technique (Li et al., 2020c). Flocculation increases the effective size of the particles before dewatering and so reduces the energy cost. Coagulation/flocculation is generally followed by gravity sedimentation which is a low-cost method of harvesting microalgae. While coagulation involves adjusting the pH or adding an electrolyte, flocculation uses cationic polymers that are added to the broth (Pei et al., 2021). Liquid biphasic flotation system is a novel method of biomolecules extraction. The system is an integration of aqueous two-phase system with mass transfer mode of solvent sublation, which has been used in the downstream processing of microalgae biorefinery (Aron et al., 2022).

After harvesting, the dewatered cake is usually dried to improve the efficiency of the downstream processes (e.g., lipid extraction) (Najjar and Abu-Shamleh, 2020). Recent technologies have shown that biofuel production from algal biomass may not be energy efficient because the production process consumes more energy than that produced by combusting the resulting biofuel. The harvesting of small algae (usually between 10 and 30 μm in diameter) is laborious and the cheaper press method of extracting oil from oilseed plants is not applicable with algae, which adds to the production cost (Saengsawang et al., 2020). Extraction of the substrates (lipids and sugars) also requires rupturing of the cell walls through an energy intensive process, depending on the algal strains (Dharmaprabhakaran et al., 2020). Lowering harvesting costs is thus important for the sustainable production of micro-algal biomass. Optimizing the method of harvest depends on the characteristics of the alga and the nature of the end product. Harvesting half of algal biomass and allowing it to double again before each subsequent harvest has proven difficult to manage (Hirooka et al., 2020). To date, most of the techniques used to harvest algae have drawbacks, such as costs of operation that may be high, and low efficiencies producing a relatively poor quality product: mechanical processes involved in sedimentation, centrifugation, and filtration can result in cell rupture, leading to leakage of cell content and a low quality (Bošnjaković and Sinaga, 2020). It is suggested that microbes should be engineered to perform direct photosynthetic and conversion of carbon by efficient nutrients and light energy capturing to produced algal biofuel and other industrial product.

## 3 Biofuel production from halophytes

**Figure 3:**
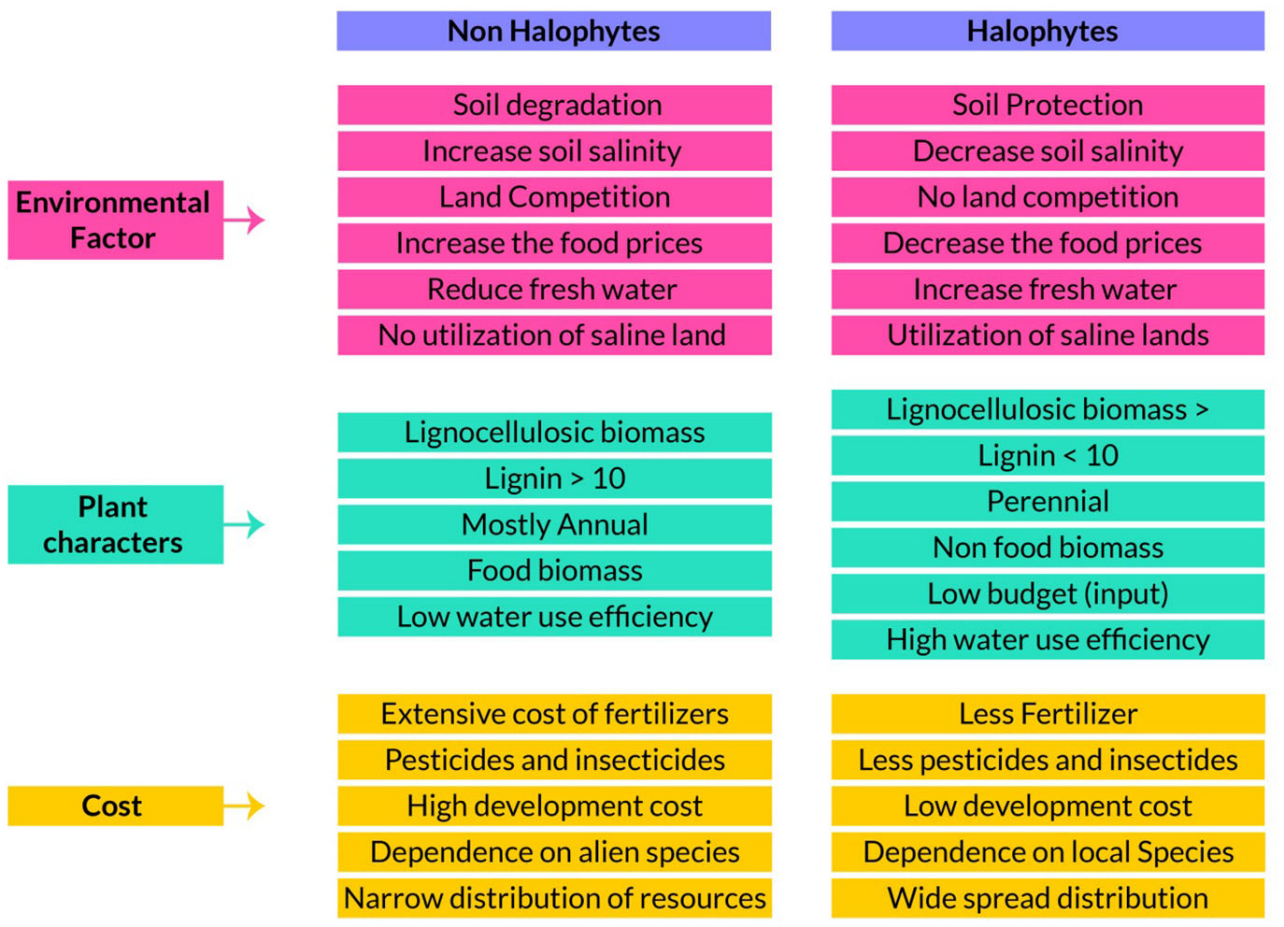
Comparisons of halophytes over non-halophytes as environment factors, plant biomass compositions and their cost effectiveness as energy source.

Halophytes appear an ecologically and economically feasible alternative to agricultural crops for biofuel production (**Figure 2**), especially in arid and semi-arid regions, because of their ability to survive in saline habitats and tolerate extremes of temperature, high irradiance, and scarcity of water (Halat et al., 2020). **Table 8** and **Figure 3** illustrate the biofuel properties generated from halophytes and glycophytes. Conversion of lignocellulosic biomass to ethanol has been widely studied in past decades. New technologies are being proposed for lignocellulosic ethanol production, which includes mild torrefaction (is a mild form of pyrolysis at temperatures typically between 200 and 320°C). Different processing methods have also been proposed after a pretreatment step, which include separate hydrolysis and fermentation, simultaneous saccharification and fermentation, simultaneous saccharification and co-fermentation and consolidate bioprocessing. In consolidate bioprocessing, lignocellulosic materials are depolymerized into sugars and simultaneously enzymes that convert the sugars to ethanol or other products (Zoghlami and Paës, 2019). Brethauer and Wyman (2010) achieved 67% ethanol yield by dilute acid pretreatment of wheat straw using natural strains of microbes. For ethanol production, organosolv pretreatment using ethanol is preferred, as the ethanol used is recovered during distillation, when the final pure product is obtained (Liu et al., 2019).

**Table 8:** Lignocellusic biomass (cellulose, hemicellulose and lignin composition of different plant feedstocks.

Species	Example	Cellulose	Hemicellulose	Lignin	References
Edible crops	Sugar beet	20.0	25.0	20.0	(Câmara-Salim et al., 2021; Turcios et al., 2021)
	Sunflower	25.0	17.0	17.0	
	Maize	33.8	25.4	8.6	
	Corn stover	38.0	28.0	7.0	
	Alfalfa	34.4	6.7	7.2	
Halophytes					
	*Halopyrum mucronatum*	37.0	28.7	5.0	[31–33]
	*Panicum turgidum*	28.0	28.0	6.0	
	*Phragmites karka*	26.0	29.0	10.3	
	*Typha domingensis*	26.3	38.7	4.7	
	*Desmostachya bipinnata*	26.7	24.7	6.7	

**Figure 4:**
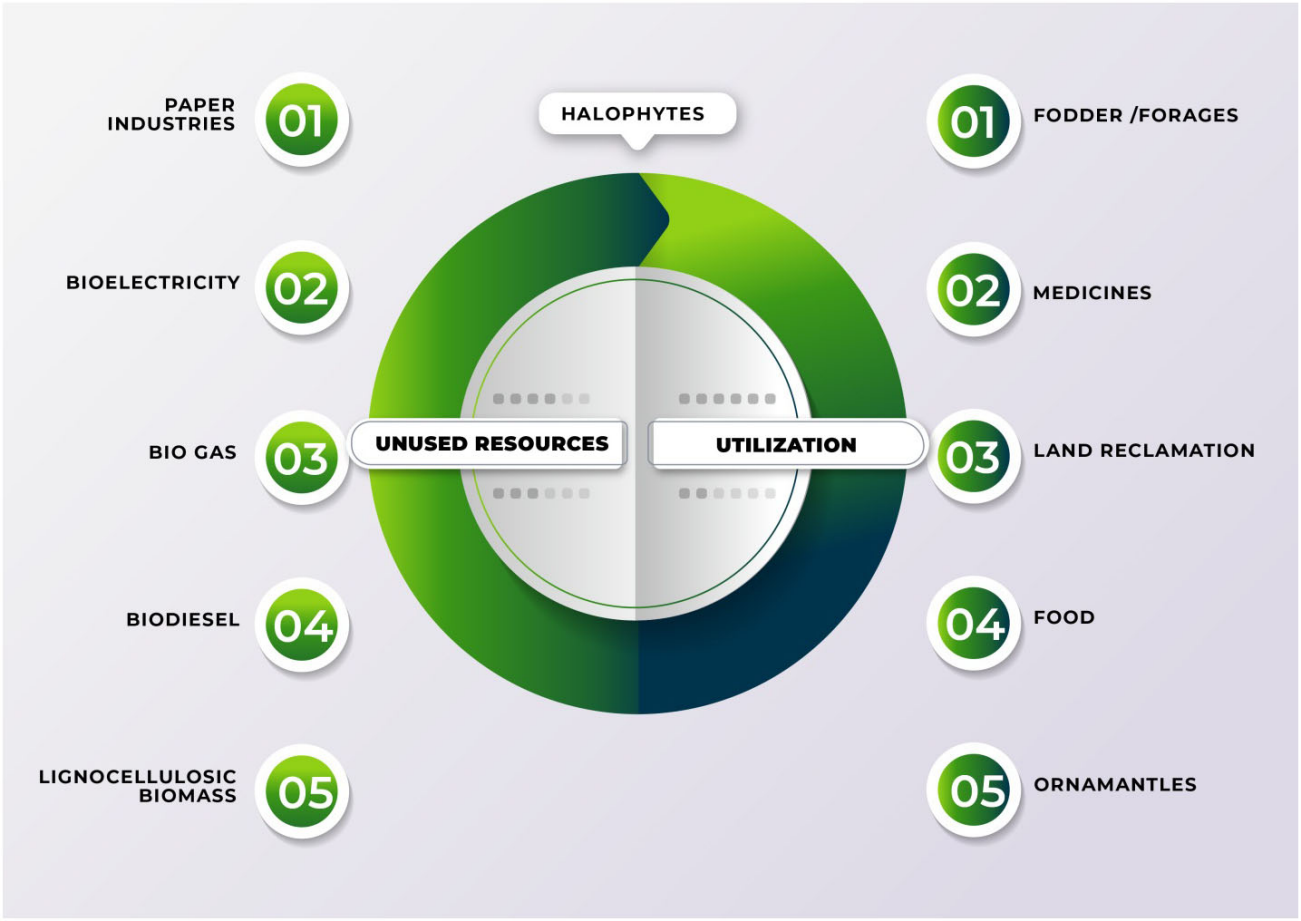
Application of salt resistant plant for different industrial purposes using saline land and brackish water.

**Figure 5:**
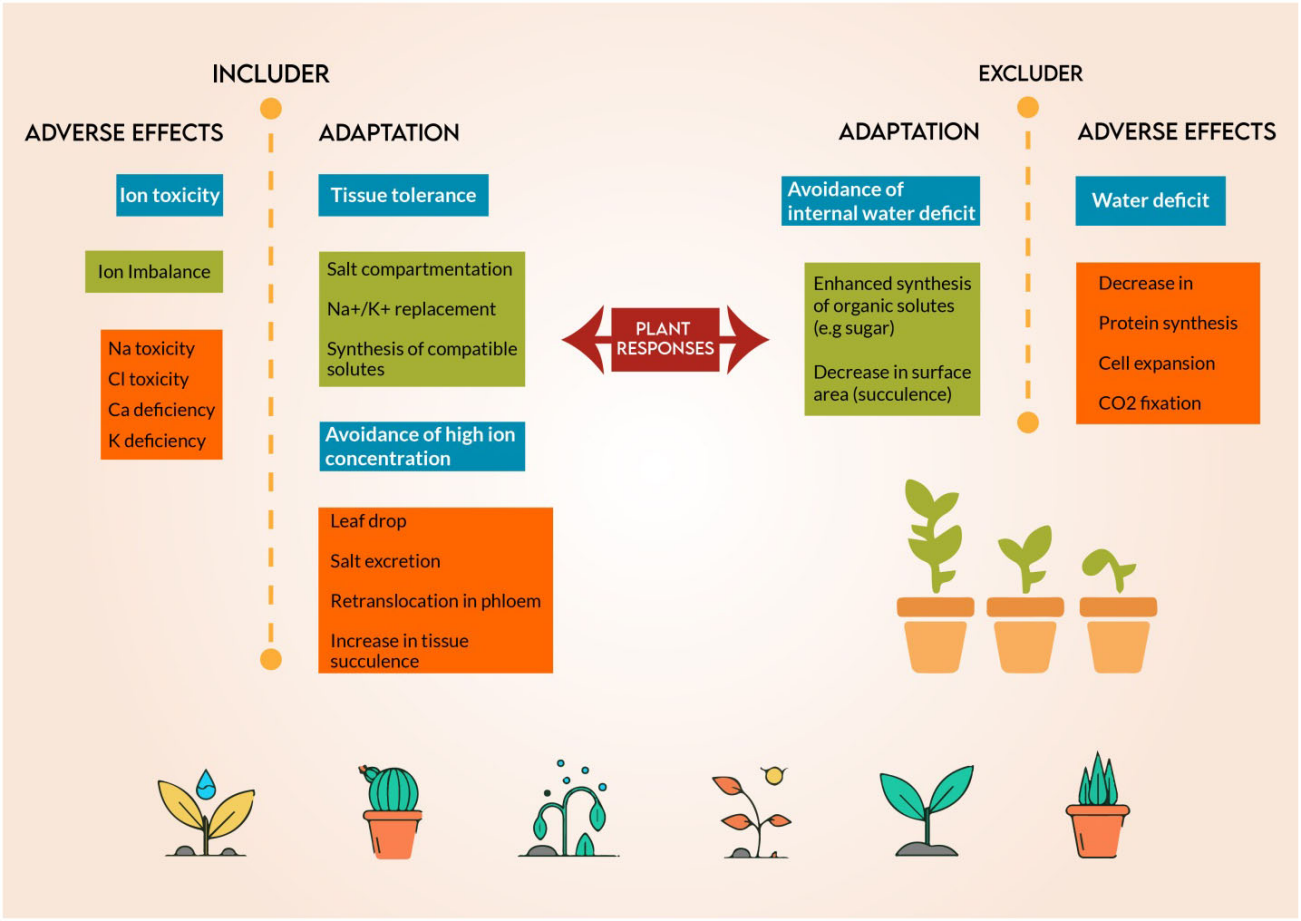
Salt tolerance machanisms and plant adaptaion of salt resitant plants to grow and complete their life cycle under saline medium.

Using plants has particular potential especially in areas where large populations of diverse halophytic species already grow and can be cultivated using saline water for irrigation without using existing arable lands or clearing forests to open new lands (Xu et al., 2020b). By providing a cover on barren lands, halophytes can reduce soil erosion and reduce greenhouse gases by C-sequestration; some halophytes are potential sources of oil seeds (Abideen et al., 2015b); others have value in medicines and various other purposes (see **Figure 4**) (Abideen et al., 2012; Abideen et al., 2014b; Yasin Ashraf et al., 2020). Many perennial halophytic grasses produce enough lignocellulosic biomass under saline conditions to warrant conversion into biofuel (Munir et al., 2021). In our preliminary study, *Halopyrum mucronatum*, *Desmostachya bipinnata*, *Typha domingensis*, *Phragmites karka* and *Panicum antidotale* emerged as suitable bioethanol candidates among halophytic grasses of coastal region of Pakistan (Toqeer et al., 2018). These species show high growth rates reaching up to a meter in height in 4-5 weeks and therefore can compete with conventional edible biofuel crops (Tipnee et al., 2015). These halophytes not only accumulate high biomass but also contain low lignin with high cellulose and hemicelluloses contents, which can make the hydrolysis of their biomass more efficient than that of the conventional biofuel crops (**Table 8**). Similarly halophytic plant species like *Salicornia fruticosa*, *Cressa cretica*, *Arthrocnemum macrostachyum*, *Alhagi maurorum*, *Halogeton glomeratus*, *Kosteletzkya virginica* and *Atriplex rosea* appear to be promising biodiesel candidates based on the quality and quantity of seed oil (Abideen et al., 2015a). Further surveys are needed to identify other halophytes that have the potential to grow satisfactorily under saline/arid conditions and can be harvested for several years without replanting, thus making them more economical to grow than annual species. However, while producing biofuel from halophytes is attractive, halophytes will only serve as a supplement to existing sources and need research to solve various problems discussed below that might occur if adopted for industrial scale biofuel production. The steps of biofuel conversion from wild halophytic plants are described in detail in **Figure 5**.

### Ecological constraints

Experimental and circumstantial evidence proves the advantages of halophytes over the present day glycophytic crops in tolerating salinity stress. However, systematic research on halophytes has gained momentum only during the last 40-50 years and shows wide diversity in growth response to salt. Some halophytic seeds may germinate better in the absence of salinity but as plants, many require salt for optimum growth (Flowers and Colmer, 2015): the optimal growth of a number of halophytes, particularly dicotyledonous species, was obtained in about 200 mM salinity (Flowers et al., 2010). Halophytic grasses like *Halopyrum mucronatum*, *Aeluropus lagopoides*, *Sporobolus ioclados* show higher growth under fresh than salt water conditions (Abideen et al., 2014a). *Phragmites karka* and other halophytic grasses like *Phragmites australis*, *P. communis*, *Spartina maritima* and *Pennisetum clandestinum* show reduced growth as the concentration of NaCl increases (Faustino et al., 2020). *Arthrocnemum macrostachyum* exhibited extreme salt resistance by surviving in up to 1000 mM NaCl salinity (Gulzar and Khan, 2008).

To survive in saline conditions, plants must adjust osmotically to the low water potentials of their growth media by accumulating solutes in their organs. On average, dicotyledonous halophytes have higher ion concentrations in their above-ground biomass than do monocotyledonous halophytes, of which the family with most salt-tolerant species is the Poaceae (Santos et al., 2016). Since efficient biomass saccharification and pyrolysis depends upon a low salt load in the foliage, salt tolerant grasses are more suitable than dicotyledonous halophytes because of their ability to restrict ion uptake at the root level and reduce salt buildup in above-ground parts (Wang et al., 2020a). But this advantage of being a better feedstock carries the associated trait of decreasing growth with increasing root zone salinity. Combining a salt excluder grass for use as biofuel feedstock and a salt accumulator in the same saline land to reduce root zone salinity may offer a partial remedy but needs further study (Huang et al., 2020). Responses to saline substrates need to be analyzed on all halophytic species with potential as sources of biofuel and to uncover the mechanism of salt resistance and ascertain the optimum level of salinity to improve productivity and identify suitable candidate(s) for use (**Figure 3**) (Lu et al., 2020). It is worth mentioning here that research on the improvement of salt tolerance of conventional crops for the benefit of mankind has been attempted over thousands of years but with little significant progress; work on the domestication of halophytes is still very limited (Yeo et al., 1990).

### 3.1 Availability of seeds

Availability of good quality seed is a basic requirement for plant production, which is critical in the case of halophytes because, unlike conventional crops, seeds of halophytes are rarely available commercially but have to be collected from the wild populations (Lombardi and Bedini, 2020). Availability, collection and multiplication of seed is labor intensive and costly (Bhatt et al., 2020). The role of the plant breeding industry is not only to produce adequate quantity of seeds but also bring desirable changes in its characteristics, keeping in consideration the end use (Munir et al., 2020). Support will be required for examining the quality and type of seed for a particular region. This will require capacity building of public institutes to produce breeder seeds and to conserve genetic resources in seed production as well as harmonization of policies and regulatory frameworks for large-scale planting. Very large nurseries are needed requiring technical know-how and this will involve cost. Tissue culture techniques may help to meet the demand, although information in this regard is scanty. If adopted, tissue culture techniques will require hands-on practical and consultative training for interested seed multipliers (Palchetti et al., 2020). Seed coating technology can modify seed shape and size with improve delivery to sites mainly for small-seeded plants and for seeds with complex morphology (Staples et al., 2020). Seed coating of *P. spicata* improves seedling emergence and growth in crusting soil as well as improving handling and sowing efficiency of small seeds such as those of *Artemisia tridentata* (Serpe et al., 2020).

### 3.2 Agronomy and cultural practices

Crop cultivation requires detailed information about best cultural practices for optimum yield, information that is available for most of our current conventional crops. However, such information is rarely available for halophytes (Hanus-Fajerska et al., 2020). Halophytes are substantially different from other agricultural crops in their morphology, growth habit, nutrition pattern and a proper understanding of various aspects of their cultivation is required if yields are to be maximized. For instance, detailed information is needed about the planting density for optimum growth, fertilizer requirement, irrigation scheduling and harvesting interval (Andreotti et al., 2020). Chemical testing of soil has long been an accepted agricultural management practice to assess soil fertility, and avoid excess fertilizer application or pollution of the environment especially in deserts with very deep water tables (Koyro, 2003). For coastal regions, this information is generally missing for halophytic plants including those meant for use as biofuel crops. Interpretations and fertility recommendations based on soil analyses and the information on cropping systems, tillage practices, soil texture and structure and manure use can contribute to increased efficiency of biomass production in saline lands (Maciel et al., 2020). Currently, the amount of land devoted to growing crops for energy is only 0.19% of the world’s total land area and only 0.5–1.7% of global agricultural land. The wasted saline land could be used for halophyte cultivation which can reduce land competition and freshwater resources so there will be no competition with the cultivated lands.

### 3.3 Knowledge of plant diseases

Losses from insects and diseases range from 9 to 16% in major field crops (rice, barley, wheat, maize, potato, soybean, cotton) (Ahmad et al., 2020) and are a significant constraint to crop production that stands between the rapidly growing world population and starvation. Research on diseases in halophytic plants is limited but needs to be pursued to find suitable control measures using methods available in conventional agriculture (Tıpırdamaz et al., 2020). The techniques of molecular biology may be helpful in analysis of gene expression for responses to different biotic and abiotic stresses and potential trade-offs. Seed coating with predator repellents can reduce seed consumption by rodents (Carter et al., 2020). Seeds coated with salicylic acid improved growth of *Austrostipa scabra*, *Microlaena stipoides*, and *Rytidosperma geniculatum* as well as survival. Biological control is a very promising strategy to control plant diseases. *Coniothyrium minitans* and *Sporidesmium sclerotivorum* are used to control diseases caused by *Sclerotinia* sp. *Coniothyrium minitans* based products are available in the European market (Patel et al., 2021). *Pseudomonas fluorescens*, which produces toxins or secondary metabolites such as siderophores, phenazines and cyanide, can be used against *Gaeumannomyces graminis* and *Chalara elegans* (Martin-Rivilla et al., 2020). However, there are no such examples for halophytes.

### 3.4 Variable seed germination and propagation

Halophytes are plants of saline habitats that grow under conditions with variable stress and may change their responses rapidly between seed germination and later growth. Germination at high salinity may provide an advantage of high seedling population to start with but subsequent growth and biomass yield may not necessarily reflect tolerance to salinity during seed germination (Becerra-Vázquez et al., 2020). Seed dormancy is an important means of delaying seed germination and initiating growth under suitable conditions. Many halophytic species do not, however, possess such elaborate systems because they naturally propagate through ramets and have no ecological compulsions for seed germination. Seed priming technology is used to reduce variability in seed germination rate with constant and rapid germination within populations (Banerjee and Roychoudhury, 2018). Seed dormancy is an important means of delaying seed germination and initiating growth under suitable conditions. Many halophytic species do not, however, possess such elaborate systems because they naturally propagate through ramets and have no ecological compulsions for seed germination. Seed priming technology is used to reduce variability in seed germination rate with constant and rapid germination within populations. Priming can provide resistance to heat, moisture and osmoticum stresses (salt) (Banerjee and Roychoudhury, 2018). Seed priming improves germination in *Guazuma ulmifolia*, *Albizia saman* (Ibrahim and Hawramee, 2019), *Cedrela odorata*, *Enterolobium cyclocarpum* (Becerra-Vázquez et al., 2020), *Swietenia macrophylla* and also the performance of *E. cyclocarpum* (Pedrini et al., 2020). Seed priming improved fast emergence in seeds of *Poa fendleriana* and *P. spicata* (66–82%), while the density of *P. spicata* seedlings was 2.9-3.8 fold higher than non-treated seeds (Madsen et al., 2018).

### 3.5 Invasive potential

Biological invasions are an increasingly important threat to biodiversity and ecosystem functioning and a major component of global change worldwide. In addition to affecting ecosystems and contributing to the extinction of native species, invasive non-native species can also cause major socio-economic damage. Many species that are currently invasive were introduced without proper study of after-effects and many spread providing usually small benefits to a sector of society but with harmful (often irreversible) environmental consequences. Once introduced and established in a new region, plant species, are extremely difficult to eradicate or control. Next generation biofuel crops, whose production demands large biomasses, have the potential to become problematic and costly invasive species that if alien for an area; need to be closely watched. Characteristics like wide environmental resistance, ease of establishment, ability to re-sprout when harvested, fast growth rate, low demand or good quality water are precisely the traits that predispose species to become invasive (Hussain et al., 2020); see **Figure 3** for comparison or similarities in biofuels and non-invasive properties. For example *Arundo donax*, *Phalaris arundinacea* and *Phragmites karka* are fast growing C_3_ invasive species with tolerance to drought, salinity, and low-fertility soils (Abideen et al., 2012; Abideen et al., 2014a; Qasim et al., 2014). They should be targeted for cultivation in specific areas where they can flourish and produce lignocellulosic biomass without harming the ecosystem.

A prospect which is apparently yet to be examined is that harvesting biomass for conversion into biofuels could be used as a tool for controlling invasive species (Nayak et al., 2020). Imperata grasslands found in Indonesia could be harvested to provide biomass, and then exchanged with more productive crops (e.g. oil palm) or with native biomass feedstocks that are non-invasive. The major risk of this approach is that it could encourage planting and spread of invasive species (Duarte and Caçador, 2021). This risk could be reduced if it were managed specifically as a control strategy, with penalties for replanting. No attempt has been made to quantify actual, relative or potential invasiveness of terrestrial biofuel crops at an appropriate regional or international scale, and their planting continues to be largely unregulated. Scientist must assess ecological risks before suggesting a new biofuel crop, to avoid introducing an invasive species (Mao et al., 2020).

## 4 Comparison of algae vs halophytes as biofuel candidates: a summary

In spite of harmful environmental consequences, fossil fuels continue to be used extensively in our daily life. The recent discovery of alternate energy sources such as shale oil have raised confidence in the continued availability of fossil resources. This optimism has been strengthened further by access to deposit sites previously considered difficult to reach. Technological improvements, coupled with general inflation in prices, have been an impetus for pumping oil from depths previously uneconomical to drill. However, there are still forecasts that these developments will only delay the inevitable loss of supply for which there is need to explore other sources of energy like nuclear, wind, solar, tidal, plant biomass as potential alternates.

Commercial interest with the rising awareness for environmental and energy issues are strong catalysts for producing biofuels from non-food resources. Halophytes and algae may be suitable options for this purpose because they do not need arable land and the freshwater required for growing food crops - with additional benefit of carbon sequestration and removing greenhouse gases (GHG) from the atmosphere by consuming CO_2_ for photosynthesis during growth. The seed germination dynamics and growth of halophytes are rarely well documented but there are examples where substantial improvement in growth can be achieved by applying different growth promoting agents such as biochip, compost and growth promoting bacteria. In the case of algae, there are a few studies that help in improvement of growth parameters; dry biomass accumulation is key factor. The major advantage that growing algae has over halophytes is the faster growth rate of the former than the latter, achieved with lower nutrient requirements. Halophytes need at least one month to establish their seedling in the saline soil. After establishment they can achieve fast growth, but seed is only produced once, or rarely twice, a year. The smaller seed size of most halophytes compared with glycophytes can reduce the efficiency of biodiesel production in halophytes compare to algal blooms. However, the feedstock from halophytes could be a good source of bioethanol production compared to algae. Halophytes can produce leaf and shoot biomass on large scale and their biomass saccharification and fermentation could give more sustainable bioethanol than other bioenergy resources including algal biomass. The cultivation of halophytes for biofuel on otherwise saline barren lands could develop the use of non-agricultural lands for industrial application, although the potential for invasiveness needs care. The pond system of cultivation of algal biomass can raise environmental problems due eutrophication following discharge of nutrient-rich water to the local water resources. The exploitation of algal biomass directly from the ocean can disturb the food web, nutrient cycling and cause ecosystem damage.

The technology of converting biofuels from non-food options is potentially sound but it is not ready for instant application and needs a cautious approach with a number of obstacles to overcome before large scale adoption. The oil contents of algal biomass sounds very appealing but starting from identifying suitable algal strains and their growth optimization under particular conditions followed by subsequent processing involving harvesting, dewatering and conversion to the end product involves covering a long, tedious and underexplored terrain. Halophytes too have several problems that need to be assessed. Starting from the difficulty of availability of seed in adequate quantities for planting on a commercial scale to dearth of information about the cultural practices for optimum biomass yield and all the intermediary management steps; there are many questions. Without answers There are also many risks and uncertainties, such as variable germination, problems with propagation, plant diseases, scaling up, processing plant biomass, market demand and economic competition with bulk-produced raw materials from other conventional crops already being used. Cost effectiveness will be the prime consideration which will be compromised if halophytic plants are grown under artificially controlled conditions such as in a green house. Plants which are growing naturally near the coast or on inland degraded areas and producing suitable feedstock with saline irrigations is likely to be a better option as such systems have other important advantages like soil protection against wind and water erosion, enhancing biodiversity, creation of habitats for animals and mitigating environmental degradation.

## 5 Conclusions

In spite of projected shortages of energy and harmful environmental consequences of their production, fossil fuels continue to be used extensively in our daily life. Commercial interest with the rising awareness for environmental and energy issues are strong catalysts for the initiative of producing biofuels from nonfood resources. Halophytes and algae may be suitable options for this purpose because they do not need the arable land and freshwater required for growing food crops with additional benefit of removing greenhouse gases from the atmosphere by consuming CO_2_ for photosynthesis during growth. Potential food value of both algae and halophytes is very limited at least for the time being – except, perhaps, the use of halophytes as high-value foods and animal feed. The facilities to produce biofuel other by products such as protein and other useful substances from the post processing residue can decrease the total cost of biofuel production. It is worth mentioning that while some algal strains contain suitable forms of carbohydrates that can be fermented to bioethanol, oil in adequate quantities can be obtained from oilseed halophytes for conversion into biodiesel.

## 6 Future prospects

The oil contents of algal biomass seem very appealing but starting from identifying suitable algal strains and their growth optimization under particular conditions and subsequent processing involving harvesting, dewatering and conversion to the end product involves covering a long, tedious and underexplored terrain. The use of halophytes also has problems that need to be assessed. Starting from the poor availability of seed in adequate quantities for planting on a commercial scale to the dearth of information about the cultural practices for optimum biomass yield and all the intermediary management steps, there are many unanswered questions. There are also many risks and uncertainties such as variable germination, problems with propagation, plant diseases, processing plant biomass, market demand and economic competition with bulk-produced raw materials from other conventional crops. Cost effectiveness will be the prime consideration, which will be compromised if halophytic plants are grown under artificially controlled conditions such as in a greenhouse. Domestication of these plants can be initiated by screening collections for the best genotypes and detailed chemical analysis to judge their suitability for particular purposes. This article has highlighted some of the stumbling blocks that may lie on the way but there may be more bottlenecks to remove for successful adoption of this novel approach. It is also evident that biofuels will not meet the total demand but will only be part of the future energy mix of liquid fuel.

## Author contributions

ZA and RA: Conceptualization, Investigation, Formal analysis, Methodology, Writing – original draft. MH: Conceptualization, Investigation, Formal analysis, Methodology, Writing – original draft. TF, H-WK, AE-K, MA, and MAK: Supervision, Conceptualization, Resources, Writing – review and editing, Funding acquisition. MH: Supervision, Conceptualization, Resources, Writing – review and editing, Funding acquisition. MA: Formal analysis. MH: Formal analysis. ZA: Conceptualization, Investigation, Formal analysis, Methodology, Writing – original draft, Formal analysis. All authors contributed to the article and approved the submitted version.
